# Modeling Respiratory Signals by Deformable Image Registration on 4DCT Lung Images

**DOI:** 10.1155/2021/6654247

**Published:** 2021-10-30

**Authors:** Pham The Bao, Hoang Thi Kieu Trang, Tran Anh Tuan, Tran Thien Thanh, Vo Hong Hai

**Affiliations:** ^1^Computer Science Department, Information Science Faculty, Sai Gon University, Ho Chi Minh City, Vietnam; ^2^Department of Nuclear Physics, University of Science, Ho Chi Minh City, Vietnam; ^3^Vietnam National University, Ho Chi Minh City, Vietnam; ^4^Department of Computer Science, University of Science, Ho Chi Minh City, Vietnam

## Abstract

The lung organ of human anatomy captured by a medical device reveals inhalation and exhalation information for treatment and monitoring. Given a large number of slices covering an area of the lung, we have a set of three-dimensional lung data. And then, by combining additionally with breath-hold measurements, we have a dataset of multigroup CT images (called 4DCT image set) that could show the lung motion and deformation over time. Up to now, it has still been a challenging problem to model a respiratory signal representing patients' breathing motion as well as simulating inhalation and exhalation process from 4DCT lung images because of its complexity. In this paper, we propose a promising hybrid approach incorporating the local binary pattern (LBP) histogram with entropy comparison to register the lung images. The segmentation process of the left and right lung is completely overcome by the minimum variance quantization and within class variance techniques which help the registration stage. The experiments are conducted on the 4DCT deformable image registration (DIR) public database giving us the overall evaluation on each stage: segmentation, registration, and modeling, to validate the effectiveness of the approach.

## 1. Introduction

Nowadays, diseases of the respiratory system have been increasing because of more and more pollution in many cities. Besides, smoking cigarettes or aging also affects the respiratory tract of people. An approach to model or visualize a respiratory cycling process from 4DCT images for diagnosis is highly encouraged but still has a lot of challenges. Although researchers in this field try to investigate and solve the problem, the results are still rather limited and unsatisfied. To develop a treatment plan by the way of modeling lung movements, registration methods must be taken into account carefully.

There are many conventional approaches in 4DCT lung images, but generally, we can classify them into three types: segmentation, registration, and modeling. In lung segmentation, in 2019, Pang et al. suggested a novel automatic segmentation model using a combination of handcrafted features (gray-level cooccurrence matrix) and deep features (U-Net) [1]. In the paper [2], in 2020, Peng et al. applied two processes to extract coarse lung contours first and then refine the segmentation depending on the basis of the principal curve model. This approach is rather complicated and requires a model initialization for the process. For registration, some researchers use deep learning approaches based on the displacement field to obtain the optimal parameters [3], which must be trained with big data until reaching the optimization. Some other approaches require a landmark tracking process [4], which must be determined by specialists.

In respiratory modeling, a question in regard to performing the registration using only the lung images still needs more researches. There are only a few papers mentioned about lung modeling in computer vision fields such as the paper of Yang et al. [5] proposed using optical flow to model the motion of the lung. The registration is applied using a multigrid approach and a feature-preserving image downsampling max filter to achieve higher computational speed and registration accuracy. Ehrhardt et al. [6] suggested using the statistical modeling which gives good model result but still depends on landmarks.

In this paper, we suggest an approach using local binary pattern (LBP) and entropy error evaluation (EEE) for registration and modeling 4DCT images into a breathing signal without using any landmark. LBP descriptor is a grayscale and rotation-invariant operator. It is not affected by rotation and variation of the images. The 4DCT lung images have dark and light areas that look like grayscale images. LBP can run faster than other descriptors and extract relevant features for the lung [7]. Then, we make a visualization of the output signal. It will help a doctor easily track or monitor a patient respiratory process for an accurate treatment plan. The segmentation, registration, and modeling stages will be described in detail. Firstly, the 4DCT images include inhale and exhale states. The images for the exhale state are segmented and served as the reference model. Secondly, images that belong to different respiratory phases from a given anatomical position are aligned with each other. The accuracy of lung segmentation is very important for registration and modeling. Minimum variance quantization (MVQ) and within class variance (WCV) methods are applied for segmentation effectively and precisely.

## 2. 4DCT Data Structure Exploratory

Generally, in a single scan, a 4DCT dataset includes about 700 to 1500 computer tomography (CT) images. Each image has two dimensions corresponding to the width and height of the image. The third dimension is the order number of slices, which is scanned at a certain defined interval along the patient's body. The last dimension is phases of scanning time.

Deformable image registration (DIR) is an emerging technology with diagnostic and therapeutic medical applications. DIR algorithms were first developed in computer vision research to estimate motion by warping a source image onto a target, producing an estimated image that visually appeared like the target image.

In this research, the 4DCT dataset was acquired as a part of the standard planning process for the treatment of thoracic malignancies at The University of Texas M. D. Anderson Cancer Center in Houston and offered by DIR-LAB [3]. In 4DCT imaging, thoracic movements are monitored by a Varian Real-time Position Management (RPM) system during the CT scan. The RPM system divides the complete respiratory cycle into ten phases, from 0% (phase T00) to 90% (T90) at 10% intervals, where 0% corresponds to the end inspiration [8]. Then, the reconstructed CT images are sorted into the ten phases based on the temporal correlation between the RPM respiration data and the CT data acquisition time of each image. The dataset has the following structure:

- First and second dimensions: 256 × 256 images
- Third dimension: 92 slices from the top to the bottom of a lung with 2.5 mm slice spacing
- Fourth dimension: phases of time from T00 to T90

**Figure 1:**
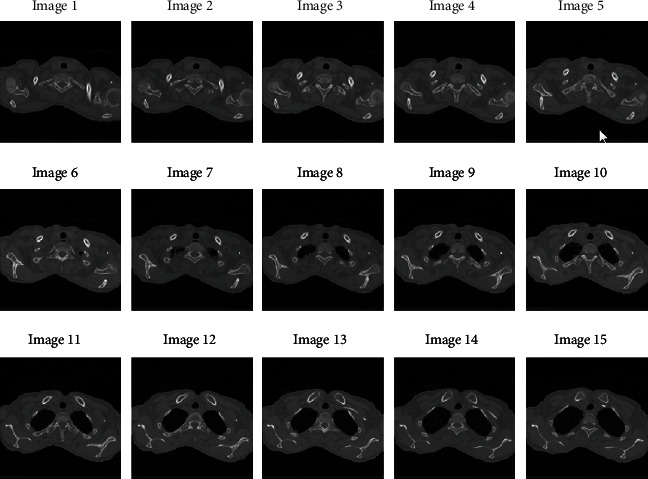
The representation of 4DCT DIR database from slides 1 to 15 in phase T00.

**Figure 2:**
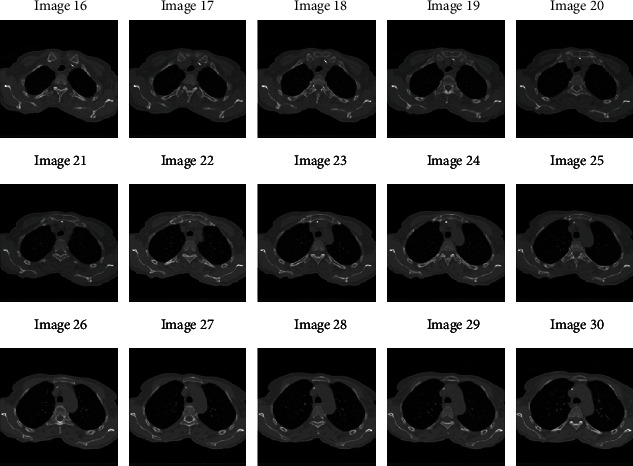
The representation of 4DCT DIR database from slides 16 to 30 in phase T00.

Figures 1 and 2 demonstrate a part of the dataset along the third dimension from slice 1 to slice 30 in phase T00.

## 3. Lung Segmentation and Artifact Removal

The process of segmentation has two steps. The first one is artifact removal, and the second one is lung segmentation.

**Figure 3:**
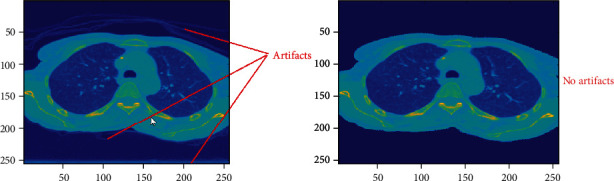
The before and after resultant of lung image in artifact removal.

*Step 1 (artifact removal)*: because the outside area of the lung and body region contains some artifacts that might affect segmentation result, the body and the lung area from the image should be extracted. To enhance the virtualization of the artifacts, the original 4DCT images are converted from grayscale to color as shown in Figure 3. Then, the minimum variance quantization method [9] is applied to cluster image pixels.

Minimum variance quantization associates pixels into groups based on the variance between their pixel values. For example, a set of blue pixels might be grouped together because they have a small variance from the mean pixel value of the group. In the lung image, the region of interest is the group of pixels at the center of the image, which contains those representing the lung. By focusing on this group, all artifacts outside the body part could be removed. These artifacts come from the lighting of background objects outside the patient's body in a scan.

**Figure 4:**
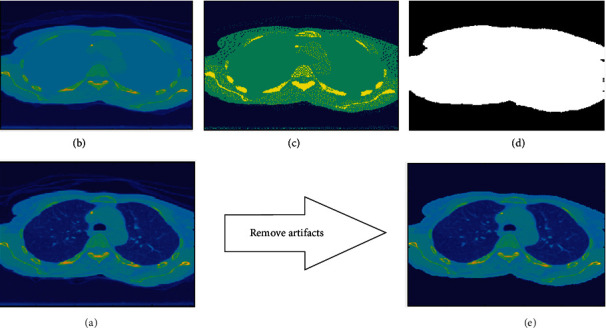
(a) Original image with artifacts. (b) Image after filling holes. (c) Image with three indexes after applying minimum variance quantization. (d) Select the maximum index region, and fill holes. (e) Segmented image with only the index region from the previous step.

In general, minimum variance quantization can be replaced by other clustering methods such as *K*-means, *K*-nearest-neighbor (KNN), and expectation maximization (EM). By comparing their results, we decide to use the minimum variance quantization for artifact removal. The details of this step are demonstrated in Figure 4 and described in Algorithm 1.

*Step 2 (lung segmentation)*: after removing artifacts, we need to segment two lungs from the image. The segmented result allows a comparison between phases and determining the inhalation and exhalation phases in a breathing cycle.

Within class variance method by Otsu [10] was applied to separate the foreground and background regions from the input image. Otsu's thresholding method involves iterating through all the possible threshold values and calculating a measure of spread for pixel values from each side of the threshold. The aim is to find the threshold value where the sum of foreground and background spreads is at the minimum.

**Figure 5:**
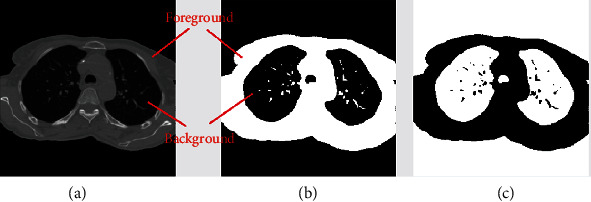
(a) Original image. (b) Foreground and background separation by Otsu threshold. (c) The complement of the (b) result.

**Figure 6:**
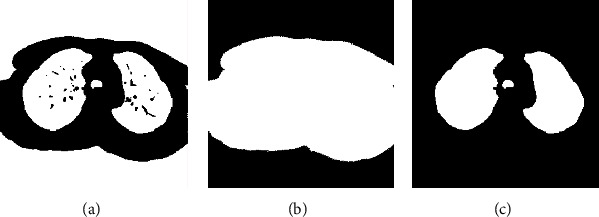
(a) The complemented binary image. (b) The body binary image. (c) The result after multiplication of two binary images (a) and (b).

**Figure 7:**
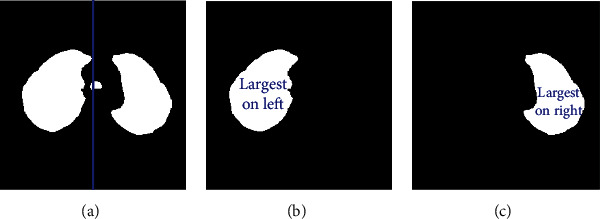
(a) Center line based on the center point of the body. (b) The left lung is the largest region on the left. (c) The right lung is the largest region on the right.

**Figure 8:**
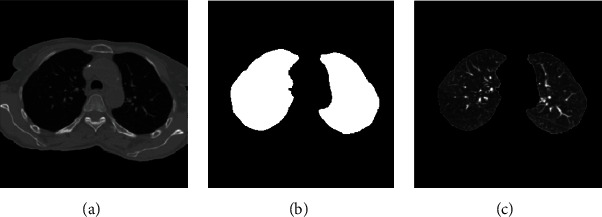
(a) Original image. (b) The binary image of two lungs. (c) The segmented two lungs.

**Figure 9:**
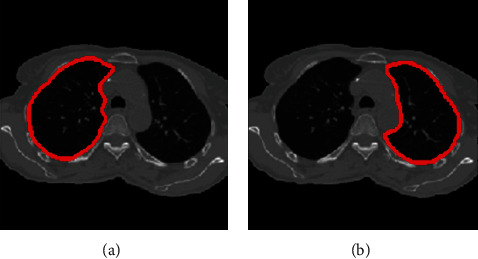
Left and right lung segmentation and highlight.

We perform the following steps to segment left and right lung areas. First, we make the complement of the binary image. Two lungs will be represented in the complemented image (in Figure 5). Second, the body region (i.e., the outline of the patient's body) is multiplied with the complemented image to obtain the regions of two lungs (in Figure 6). Note that there remain some unexpected regions besides lung areas. Third, the center point of the body image is used to segment the two lungs exactly. The left and right lungs are now on the opposite sides of the center point and represented by the largest white regions in the multiplied image (Figures 7–9). Detailed calculation steps are described in Algorithms 2 and 3.

## 4. Deformable Image Registration

In this step, we need to locate the position of a slice belonging to one phase to match with another slice in a different phase. By matching the slice of two phases, we can register these slices and reconstruct the exhalation and inhalation phases.

### 4.1. Texture Matching by Local Binary Pattern

Before applying local binary pattern (LBP) [11] to the lung image, a LBP descriptor should be determined. First, we convert the input color image to grayscales, since LBP works only on grayscale images. For each pixel, we calculate the LBP value using its neighborhood. After calculating the LBP value of the pixel, we update the corresponding pixel location in the LBP mask, which has the same matrix dimension as the input image, with the calculated LBP value.

**Figure 10:**
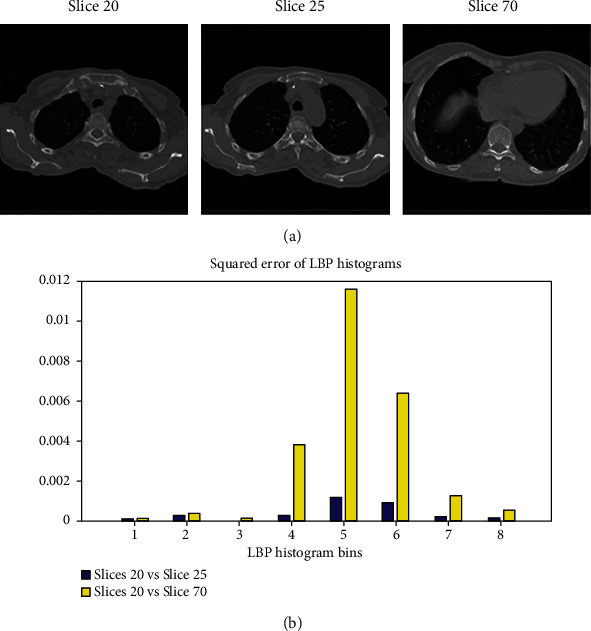
(a) The lung presentation in slices 20th, 25th, and 70th. (b) The squared error of LBP (formula in Appendix in Algorithm 4) between slices. Slices 20th and 70th have lower squared error than slices 20th and 25th in all bins. This means that the slices 20th and 25th are in the same respiratory stage, while the slices 20th and 70th are not in the same stage.

Around each pixel, there are 8 neighboring ones. If the central pixel value is greater or equal to the value of a given neighboring pixel, the corresponding value in the binary array is set to 1, otherwise is set to 0. After calculating the LBP mask, we construct the LBP histogram. The LBP mask values range from 0 to 255, giving the LBP descriptor size of 1 × 256. Then, the LBP histograms are normalized for comparison.  Figure 10 illustrates the application of LBP in comparing two contexts from two images.

Next, we apply the LBP to create a metric for a comparison of slices in different phases. LBP will return a pair of slices with the most similarity in texture. By subtracting the LBP of two slices, we can extract the LBP error metric for the registration process.  Algorithm 4 describes the method of calculating local binary pattern error rate.

### 4.2. Registration Decision by Entropy Error Measurement

Entropy is a measure of the disorder level of a system [12]. The more the disorder, the higher the entropy of the system. Two slices with the same entropy will have a high probability to be in the same registered position. Although the LBP helps us to make the texture matching between slices, in some specific cases, we can get wrong results or are unable to decide which slice in two or three slices having the similar LBP metrics. Therefore, entropy can support our decision in registration. By subtracting the entropy of two slices, we can get the entropy error metric for our registration process.  Algorithm 5 shows the steps to calculate the entropy error rate of the images.

## 5. Modeling Respiratory Signals of Inhalation and Exhalation

In the process of modeling the respiratory of signals of inhalation and exhalation, we apply LBP and entropy methods in Section 4. The following is an example of registering phase T30 to phase T00 and decide if the checking image belongs to inhaling or exhaling stages.

**Figure 11:**
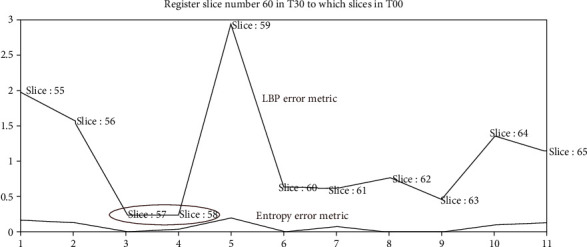
The registration from one image with the whole phase using LBP and entropy error metric.

**Figure 12:**
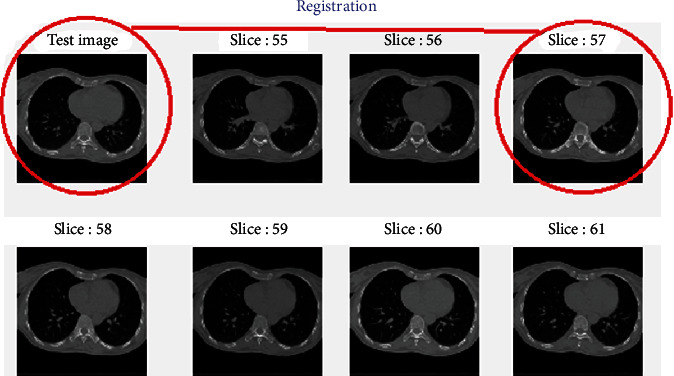
The registration from one image with the whole phase in visualization.

For example, for slide 60 in phase T30, we need to find the most similar slice in phase T00. Three steps are performed as follows (Figures 11 and 12 and Algorithm 6):

- Considering only the slices from 55 to 65 in T00 (the margin is 5 slices)
- Comparing the LBP error metrics, there are two slices 57 and 58 with minimum LBP error metrics. We need to determine which one could be registered for slice 60 in phase T30
- Comparing the entropy error metrics of the slices 57 and 58, we see that the slice 60 in T30 can be registered to the slice 57 in T00 because the entropy error metric of slice 57 is less than that of slice 58
- After registration, we notice that the process from phases T00 to T30 is the inhalation stage of the breathing process of a patient

## 6. Evaluation of Experimental Results

### 6.1. Ground Truth Lung Segmentation Determination

The DIR database provides 4DCT lung image datasets from the phase indexes T00 to T90. In each phase, a 4DCT dataset contains 94 images which are scanned from the top to the bottom of a patient lung. However, there is no ground truth lung segmentation that is specified by a specialist. To solve this problem, the ground truth segmentation is determined based on grayscale pixel values on the boundary between the lung and body partitions.

Table 1 shows the ground truth segmentation method of some slices in phase T20. The ground truth lung segmentation has an important role in the next processes of registration and modeling. If the segmentation is not close to the real lung partition, all following calculated comparison metrics in the registration will give unexpected results.

### 6.2. Lung Segmentation Evaluation

To evaluate the quality of lung segmentation for the left and right partitions, we use Dice's similarity coefficient (DSC), which measures the volume overlap percentage. The DSC is described as

(1) $$\begin{array}{c} \text{DSC}=\frac{V_{s}\cap V_{t}\;}{\left(V_{s}+V_{t}\right)/2}.100, \end{array}$$

where *V*_*s*_ is the volume of the left (or right) experimental segmentation and *V*_*t*_ is the volume of the corresponding ground truth segmentation. The closer to 100% DSC is, the better confident and efficient the segmentation is.  Table 2 shows that the result of DSC is from 96% to 99%. The slices from 30 to 70, which are the major slices in a phase dataset, have especially high DSC values.

### 6.3. Deformable Lung Registration Evaluation

For evaluation of registration, we apply the coefficient of variation (CVar) to compare with the registration for other datasets. The formula for CVar is

(2) $$\begin{array}{c} \text{CVar}=\frac{s\;}{\bar{X}}.100, \end{array}$$

where *s* and $\bar{X}$ are the standard deviation and the mean of all registration results, respectively.

In a 4DCT dataset, not all slices contribute to the registration or modeling the respiratory phase. In general, only the slices from 30 to 70 are significant in the comparison because they have clear lung segmentation information.  Table 3 shows that lung information is trivial or undeterminable for images outside that range.

Table 4 demonstrates the coefficient of variation from the phases T10 to T90. In this experiment, the source phase is T00 and the target phase is T10 to T90. The CVars are small enough to indicate high confidence. The registration of phases T10 and T90 is more confident. The registration of T20, T60, T70, and T80 has acceptable CVars. The CVars for T30 and T40 are high but are still controllable.

### 6.4. Respiratory Signal Evaluation

Inhalation (exhalation) is a process of inbreathing (breathing). The lung becomes small (large) in the inhalation (exhalation) stage. If phase T00 is the starting of the inhalation process, the error rate of LBP and entropy will be small in the registration for T10 and T00. On the contrary, if any phase in registration to T00 has a high error rate LBP and entropy, that phase is in the exhalation stage.

**Figure 13:**
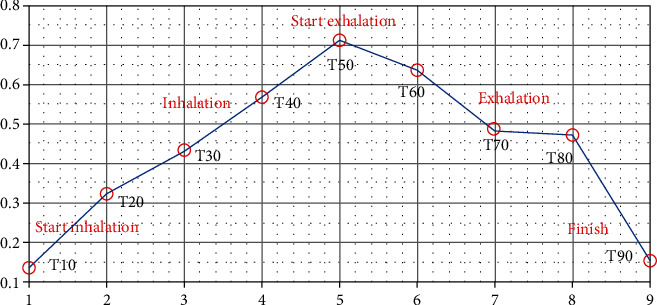
The demonstration of respiratory signal from phases T10 to T90 in registration to phase T00.

In Table 5, the sum of standard deviations of LBP and entropy error rate on each slice from 30 to 70 in each phase is calculated. LBP and entropy error rate are the appropriate metrics to represent the inhalation and exhalation of a lung. Starting from phase T00, the error rate summation increases in phase T50 and decreases in phase T90.  Figure 13, which illustrates the values in Table 5, shows that registration and modeling are successful.

**Figure 14:**
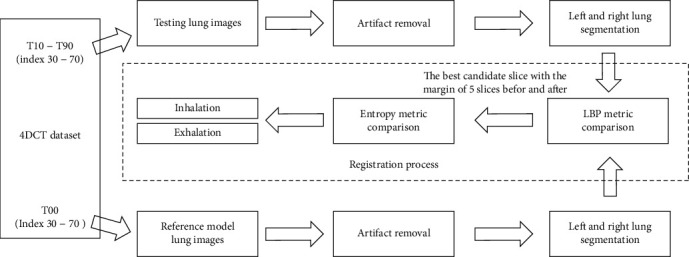
The overall framework of the proposed registration process with LBP and entropy measurements.

Figure 14 describes the overall framework for this registration step. In this workflow, the artifact removal and lung segmentation are applied for testing images and reference images. The registration process with LBP and entropy measurements is the key for checking the best candidate before giving the final decision in choosing one of the two states, inhalation or exhalation. The reference model lung images are used from the source phase T00, and the testing lung images are used from the target phases T10-T90. In each phase, the images with indexes from 30 to 70 are used because there are available lung segments in this index range.

In comparison with the learning-based approach in registration problem VoxelMorph (Balakrishnan et al.) [13, 14], diffeomorphic (Mok and Chung) [15], and DeepFLASH (Wang and Zhang) [16] registration, we have the following conclusion about advantages and disadvantages.

These approaches are applied mainly to the MRI brain images, which are more complicated with at least five segments: background, skull, white matter, gray matter, and cerebrospinal fluid. Moreover, the movements of these segments are also too difficult to track. Therefore, the authors Balakrishnan et al. and Mok and Chung propose the learning-based approach using a feature map from U-Net to minimize the loss function. Their approaches require large amounts of data for feeding and tracking in the training process.

Because we only want to model the respiratory signals, using U-Net is more complicated than necessary in the lung registration step. This is the main point of using a hybrid LBP descriptor with entropy registration in our approach. We do experiments for VoxelMorph and diffeomorphic methods with our data. The Dice measurements of VoxelMorph and diffeomorphic methods are 90% and 97%, respectively, in comparison with 96% of our proposed method. If we feed more training data, the result of VoxelMorph and DeepFLASH would be higher. Another comparison is with the DeepFLASH method, which applies the duel net with frequency spectrum domain. The Dice measurement for the DeepFLASH method is 91%. Similarly, if we continue training, the result might be improved. The advantage of our approach is that it is a fast and effective method for modeling respiratory. This method does not require more data for feeding training. We only need the reference model lung images to control the modeling.

## 7. Conclusion

There are two stages in the process of registration and respiratory modeling for the 4DCT image. The first stage, which is essential to the whole process, is lung segmentation, and the second stage is registration and modeling. If the artifacts are not removed completely, the subsequent metrics used in the registration and modeling give incorrect results. The more accurate segmentation is performed, the more accurate registration is obtained. Therefore, the minimum variance quantization and within class variance are combined for a good segmentation.

After segmentation, the LBP and entropy are applied in sequence to perform the registration. LBP can be used to find near context information between two images in different phases. Then, the entropy verifies and decides the correct registered image. If LBP and entropy are applied independently, the result becomes incorrect. Because all images in neighbor slices are similar in visualization, our method enhances efficiency of the automatic process in registration and respiratory modeling for the 4DCT datasets.

In summary, our proposed approach in modeling respiratory signals by deformable image registration on 4DCT lung images has some discriminant and promising features in comparison to conventional and deep learning approaches as follows:

- We construct a complete process from segmentation, registration, and modeling with careful selections from the minimum variance quantization method, LBP feature descriptor to entropy measurement to minimize the complexity of the process
- We still ensure the high accuracy in segmentation via DSC measurement and in registration via CVar measurement, as well as in modeling via LBP and entropy error rate
- We do not need too many images like other deep learning approaches for training data
- We can have a comparative and robust result in comparison to other traditional computer vision approaches
- The results of DSC, CVar, and entropy in segmentation, registration, and modeling can be applied as parameters for constructing loss function in deep learning approaches

Besides the above advantages, the only limitation is that our approach cannot work well if the background illumination is quite different between the reference and test images.

## Figures and Tables

**Algorithm 1:**
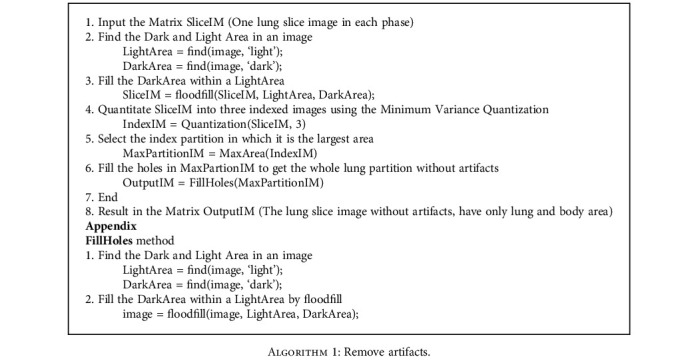
Remove artifacts.

**Algorithm 2:**
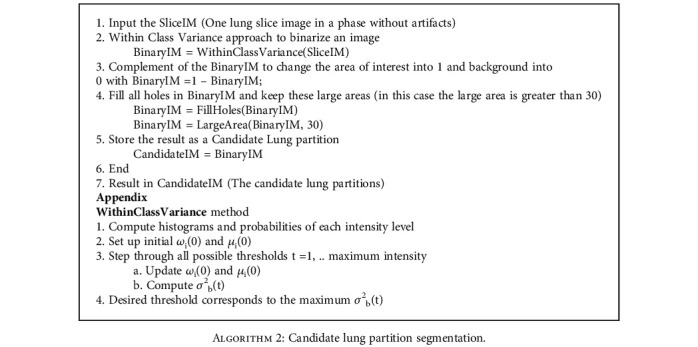
Candidate lung partition segmentation.

**Algorithm 3:**
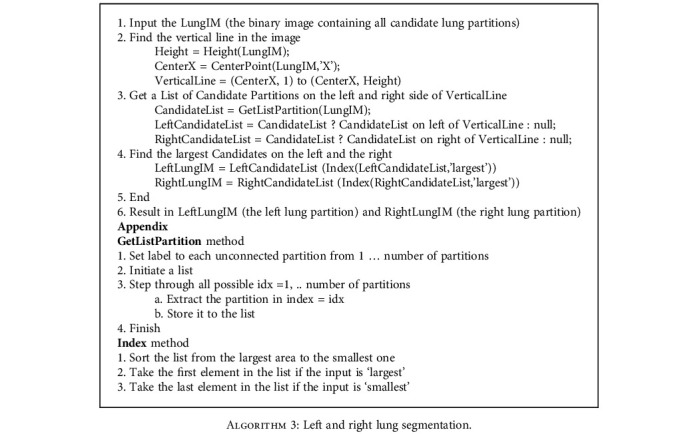
Left and right lung segmentation.

**Algorithm 4:**
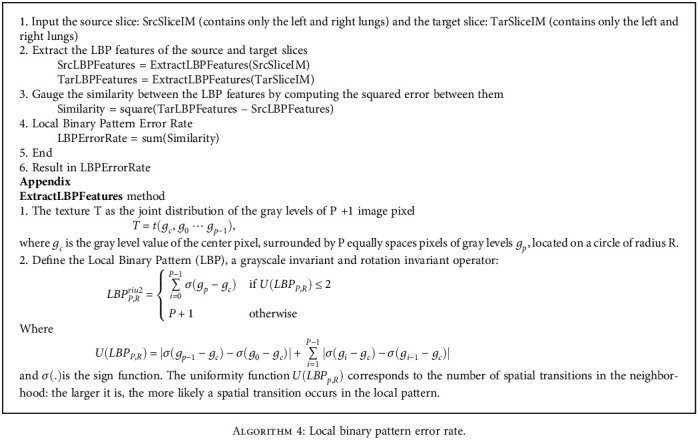
Local binary pattern error rate.

**Algorithm 5:**
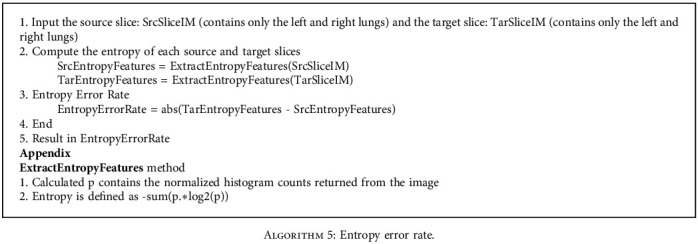
Entropy error rate.

**Algorithm 6:**
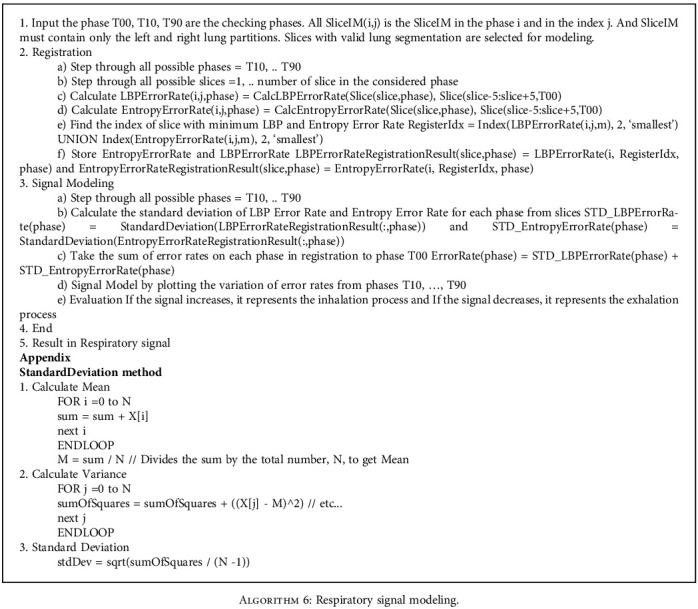
Respiratory signal modeling.

**Table 1:** The ground truth lung segmentation from phase T20.

Pixel boundary specification	Threshold	Ground truth segmentation
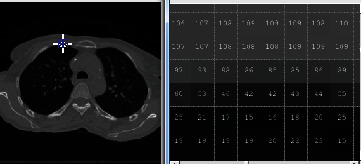	Slice 30 Phase T20 42	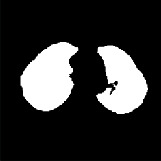
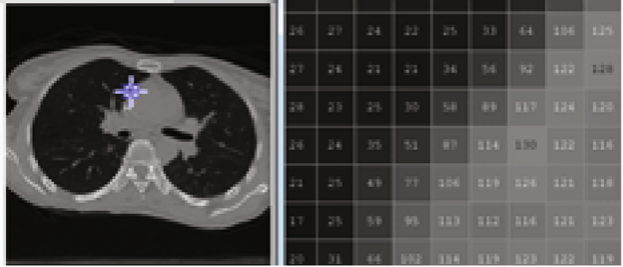	Slice 40 Phase T20 51	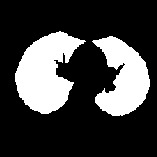
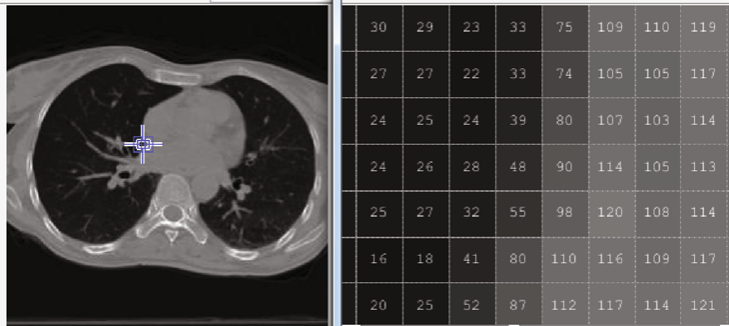	Slice 50 Phase T20 48	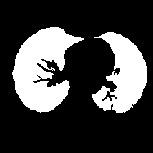
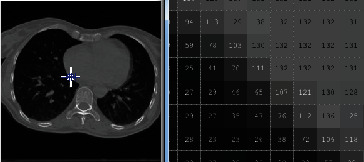	Slice 60 Phase T20 65	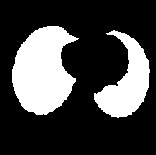
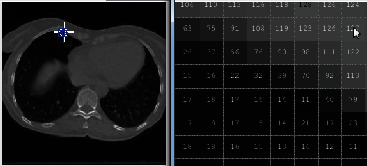	Slice 70 Phase T20 76	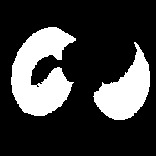

**Table 2:** The DSC measurement to evaluate the lung segmentation on ground truth in phase T20.

Slide	Original slice	Ground truth	Segmentation	DSC
Slice 30, phase T20	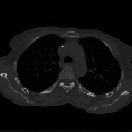	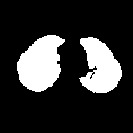	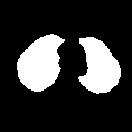	98.08%
Slice 40, phase T20	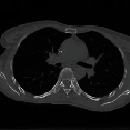	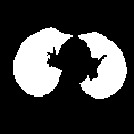	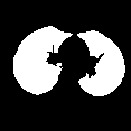	98.54%
Slice 50, phase T20	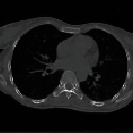	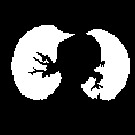	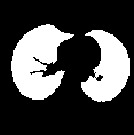	96.13%
Slice 60, phase T20	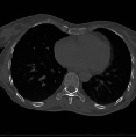	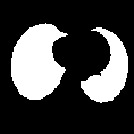	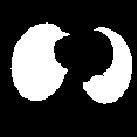	99.61%
Slice 70, phase T20	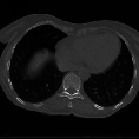	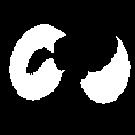	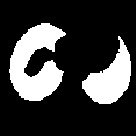	97.68%

**Table 3:** The slices from 1 to 30 and 70 to 94 are unused and insignificant for registration because of little lung information.

Slice index	Slice lung information	Remark
01	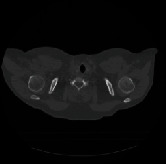	No lung information
15	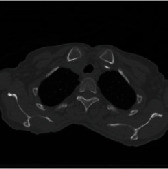	Too little lung information
80		The undetermined shape of the lung
90	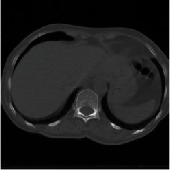	No left lung information

**Table 4:** The CVar measurement of the registration process from target phases T10 to T90 in registration to the phase T00.

Registration of the target phase to the source phase T00	Slice index range in calculation	CVar measurements	Remark
T10	Index from 30 to 70	1.6117	Confident
T20	Index from 30 to 70	1.9134	Acceptable
T30	Index from 30 to 70	2.0173	Relatively high
T40	Index from 30 to 70	2.1167	Relatively high
T50	Index from 30 to 70	1.9572	Acceptable
T60	Index from 30 to 70	1.9207	Acceptable
T70	Index from 30 to 70	1.9559	Acceptable
T80	Index from 30 to 70	1.8909	Acceptable
T90	Index from 30 to 70	1.2455	Confident

**Table 5:** The sum of LBP and entropy error rate represents the respiratory signal for slides from 30 to 70.

Registration of target phase to the source phase T00	Slice index	Sum of standard deviation of LBP and entropy error rate	Remark
T10	Index from 30 to 70	0.1370	Start inhalation
T20	Index from 30 to 70	0.3220	Inhalation
T30	Index from 30 to 70	0.4321	Inhalation
T40	Index from 30 to 70	0.5655	Inhalation
T50	Index from 30 to 70	0.7124	Start exhalation
T60	Index from 30 to 70	0.6361	Exhalation
T70	Index from 30 to 70	0.4863	Exhalation
T80	Index from 30 to 70	0.4721	Exhalation
T90	Index from 30 to 70	0.1552	Finish

## Data Availability

This research uses public data offered by DIR-LAB.
